# Role of autophagy in skin photoaging: A narrative review

**DOI:** 10.1097/MD.0000000000037178

**Published:** 2024-02-23

**Authors:** Xiaojiao Zhong, Ying Deng, Hongqiu Yang, Xiaoshuang Du, Ping Liu, Yu Du

**Affiliations:** a Medical Cosmetic Center, The Affiliated Traditional Chinese Medicine Hospital, Southwest Medical University, Luzhou, China.

**Keywords:** apoptosis, autophagy, cell proliferation, DNA damage, oxidative stress, photoaging, ultraviolet radiation

## Abstract

As the largest organ of the human body, the skin serves as the primary barrier against external damage. The continuous increase in human activities and environmental pollution has resulted in the ongoing depletion of the ozone layer. Excessive exposure to ultraviolet (UV) radiation enhances the impact of external factors on the skin, leading to photoaging. Photoaging causes physical and psychological damage to the human body. The prevention and management of photoaging have attracted increased attention in recent years. Despite significant progress in understanding and mitigating UV-induced photoaging, the precise mechanisms through which autophagy contributes to the prevention of photoaging remain unclear. Given the important role of autophagy in repairing UV-induced DNA damage and scavenging oxidized lipids, autophagy is considered a novel strategy for preventing the occurrence of photoaging and other UV light-induced skin diseases. This review aims to elucidate the biochemical and clinical features of photoaging, the relationship of skin photoaging and chronological aging, the mechanisms underlying skin photoaging and autophagy, and the role of autophagy in skin photoaging.

## 1. Introduction

Skin aging is a complex biological process that results in the gradual loss of skin function and structure under the combined action of multiple factors. It can be classified as chronological and extrinsic.^[1]^ Chronological aging refers to natural aging determined by genes and hormones, whereas extrinsic aging is caused by the cumulative effects of external factors, including environmental factors, lifestyle-related factors, systemic diseases, medications.^[2–4]^ Chronic skin damage caused by ultraviolet (UV) radiation, which is the main cause of skin aging.^[5]^ Therefore, extrinsic skin aging is commonly referred to as skin photoaging. Facial skin aging is attributed to the detrimental effects of photoaging in approximately 80% of cases.^[6]^ Prolonged or recurrent exposure to UV radiation can induce genotoxic stress, resulting in DNA damage in skin cells because of cellular senescence. This damage indirectly increases the levels of intracellular free radicals through a series of processes triggered by apoptosis. Consequently, the areas of the skin exposed to light, particularly the face and neck, may exhibit roughness, hypertrophy, dryness, desquamation, loss of elasticity, relaxation, deep wrinkles, pigmentation abnormalities, and capillary dilatation. In addition, prolonged exposure to UV radiation may lead to benign or malignant tumors.^[7]^ Approximately 90% of skin cancer cases can be attributed to UV exposure.^[8]^ The histological features of skin photoaging include the accumulation of abnormal elastin in the superficial dermis and the vasodilatation, curvature, and hypertrophy of the vessel wall.^[9,10]^ Many individuals, particularly women, use cosmetic and pharmaceutical products to prevent skin aging. The prevention and treatment of photoaging have received substantial attention from researchers worldwide. Studies have shown that autophagy plays a role in skin photoaging.^[11]^ Autophagy is a highly conserved process of cellular catabolism that occurs in eukaryotic organisms. During starvation and oxidative stress, the removal of damaged organelles via autophagy increases cell viability and maintains homeostasis within the organism.^[3,12]^ Numerous studies have demonstrated that inhibition of autophagy accelerates senescence.^[11]^ Exposure to ultraviolet A (UVA), ultraviolet B (UVB), and ultraviolet C (UVC) radiation induces autophagosome formation and upregulates autophagy markers, suggesting that dysregulation of autophagy contributes to the initiation of various dermatological disorders.^[13–15]^ However, the precise mechanisms through which autophagy contributes to the deceleration of skin photoaging remain elusive. In this narrative review, we provide a comprehensive overview of the characteristics of skin photoaging and chronological aging (Table 1),^[5,16–42]^ the mechanisms underlying autophagy and skin photoaging, and the relationship between autophagy and skin photoaging.

**Table 1:** Difference between skin photoaging and chronological aging.

	Skin photoaging	Chronological aging	Reference
Causes	UV radiation	Heredity	^[6,15–17]^
Placement	Sunlit area	Non-illuminated area	^[18]^
Relationship to age	Can be non-parallel	Parallel	^[19,20]^
Diagnostic trait			
Loss of skin elasticity	++	+	^[21]^
Skin thinning	Can be absent or thickening can be observed	++	^[20,22]^
Color	Uneven color and hyperpigmentation	No significant change	^[23]^
Wrinkles	Coarse wrinkles and leather-like appearance	Fine wrinkles	^[15,21]^
Dilated capillaries	++	−	
Sebaceous glands	Presence of nodules, cysts, and acne	No similar changes	^[5,24]^
Tumors	+++	+	^[25–28]^
Incidence	Atrophy at all levels of the skin	Abnormal, ineffective proliferative response mediated by chronic inflammation	^[16,29]^
Histological characteristics			
1. Epidermis	Irregular thickening	Generalized atrophy and thinning	^[15,21,30–32]^
Keratinocytes	Apoptosis	Decreased proliferation and differentiation	^[33]^
Langerhans cells	Decreased abundance	Decreased abundance	^[34,35]^
Melanocytes	Increased abundance	Decreased abundance	^[25,33]^
2. Dermis	Thickened	Shrinkage	^[15,31,36]^
Fibroblasts	Reduced proliferation	Decreased abundance	^[21,33,37]^
Elastin	Deformation	Degraded and reduced levels	^[6,33]^
Collagen fibers	Serious deficiency	Decreased synthesis	^[21,38,39]^
Blood vessels and skin appendages	Thickening of the vessel wall, disturbed arrangement of the vascular network, and inflammatory cell infiltration	Shrinkage	^[33,40,41]^
Preventability	Possible	Not possible	^[7,42]^

## 2. Methods

### 2.1. Selection criteria and search strategy

Peer-reviewed articles on autophagy and skin aging were searched in the MEDLINE (PubMed) and Web of Science databases from the date of database inception to August 2023. The literature search was conducted using various combinations of keywords, including “photoaging,” “ultraviolet radiation,” “autophagy,” “oxidative stress,” “DNA damage,” “cell proliferation,” and “apoptosis.” The inclusion criteria were as follows: articles with full-text access, articles published in English, and original research articles related to skin photoaging. Eventually, 123 papers were included in this review.

## 3. Results and discussion

### 3.1. Mechanisms underlying skin photoaging

Sunlight on the Earth’s surface consists of approximately 52% to 55% infrared, 44% visible, and 3% UV light.^[43]^The primary determinant of photoaging is the prolonged exposure of the skin to solar UV radiation.^[44]^ Based on the wavelength, UV radiation is classified as UVA (320–400 nm), UVB (280–320 nm), and UVC (100–280 nm).^[45]^ The extent of skin damage resulting from UV absorption depends on various factors, such as skin color, duration of exposure, geographical location in terms of latitude and altitude, seasonal variations in the intensity of UV radiation, and the specific wavelength of UV radiation.^[46]^ UVC radiation is absorbed by the ozone layer,^[47]^ whereas UVA radiation effectively penetrates the dermal layer of the skin and extends into the subcutaneous tissue layer.^[48]^ Exposure to UVA radiation induces the generation of reactive oxygen species (ROS), resulting in the impairment of DNA and other biomolecules. ROS are considered the primary catalysts driving alterations in the extracellular matrix of the dermis in photodamaged skin.^[46]^ Recent studies have indicated that UVB radiation effectively penetrates the epidermal and dermal epithelial layers, resulting in alterations in the dermal connective tissue via intercellular communication between the epidermis and dermis.^[49]^ The mechanisms underlying photoaging caused by UV (mainly UVA and UVB) radiation are mainly related to oxidative stress, DNA damage, inflammation, immunity, apoptosis, telomere shortening, and hormonal changes.^[50]^ Oxidative stress resulting from the imbalance between ROS production (caused by internal and external stimuli) and antioxidant defenses has been identified as the primary pathogenic mechanism underlying skin photoaging.^[51]^ Excess ROS can induce the activation of mitogen-activated protein kinases, thereby triggering the activation of c-Jun N-terminal kinase, extracellular signal-regulated protein kinases, and other signaling pathways.^[52–54]^ Consequently, these activated factors induce the activation of the transcription factor activator protein 1. Excessive activation of activator protein 1 can result in the upregulation of matrix metalloproteinases (MMPs),^[55]^ which are a group of enzymes that can degrade almost all types of proteins in the extracellular matrix, including collagen and elastin. In addition, excessive accumulation of ROS can trigger the activation of nuclear transcription factor signaling pathways, leading to the upregulation of inflammatory factors such as interleukin-1, interleukin-2, and tumor necrosis factor alpha. These inflammatory factors facilitate the degradation of collagen, eventually contributing to the development of skin photoaging.^[56,57]^ Antioxidants such as catalase, hydrogen peroxidase, superoxide dismutase, and glutathione peroxidase are produced to scavenge ROS and alleviate oxidative damage through enzymatic degradation, thereby reestablishing or maintaining redox homeostasis.

**Figure 1.**
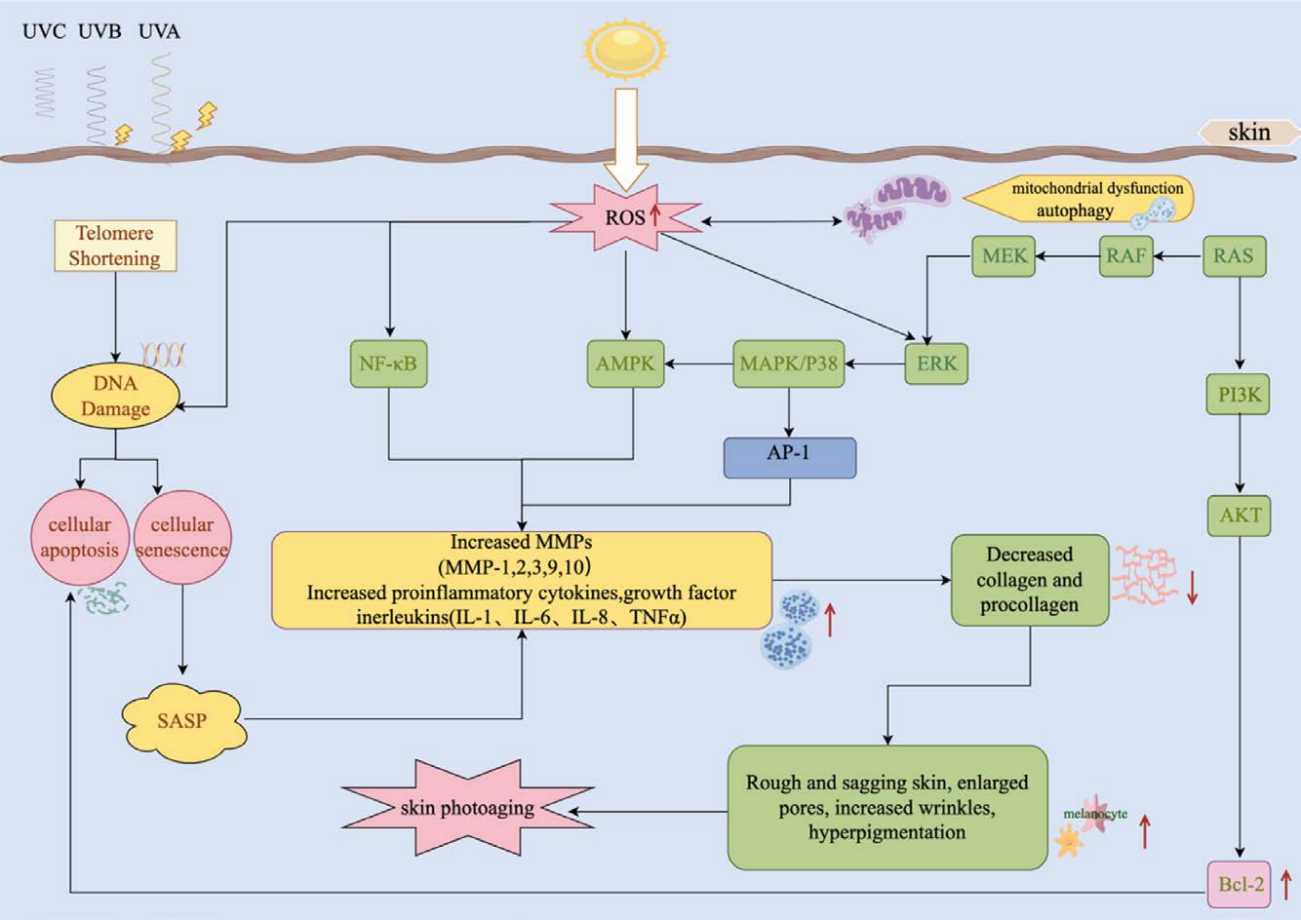
Mechanisms underlying skin photoaging (drawn using Figdraw). Arrows indicate activating effects. Ultraviolet (UV) radiation directly and indirectly disturbs the in vivo homeostasis of the skin, induces oxidative and mitochondrial stress, and exacerbates skin photoaging. The panel in the image depicts the aberrant function of multiple signaling pathways and factors that maintain cellular health. AMPK = 5´AMP-activated protein kinase, AKT = AKT serine/threonine protein kinase, AP-1 = activator protein 1, Bcl-2 = B-cell lymphoma-2, ERK = extracellular signal regulated protein kinase, IL-1 = interleukin-1, MMPs = matrix metalloproteinases, MAPK = mitogen-activated protein kinase, NF-κB = nuclear factor kappa light-chain enhancer of activated B cells, PI3K = phosphatidyl inositol 3 kinase, ROS = reactive oxygen species, Ras = Rat sarcoma protein, Raf = mitogen-activated protein kinase, SASP = senescence-associated secretory phenotype, TNF-α=tumor necrosis factor alpha, UVA = ultraviolet A, UVB = ultraviolet B, UVC = ultraviolet C.

UV radiation can cause skin damage directly by penetrating biomolecules and indirectly by increasing the production of ROS and reactive nitrogen species.^[58]^ Under pathological conditions, mitochondria are targeted by ROS and reactive nitrogen species and play a key role in controlling apoptosis. Mitochondrial proteins are more susceptible to oxidative damage caused by UV radiation-induced ROS production. The excessive accumulation of oxidative lipids and redox-active proteins may lead to mitochondrial dysfunction. Damaged mitochondria can generate a large amount of ROS, which act as signaling molecules to activate autophagy. Autophagy removes damaged mitochondria to inhibit ROS production and prevent further oxidative damage. Mitochondrial autophagy plays a key role in maintaining mitochondrial quality control by selectively removing senescent and damaged mitochondria through specific phagocytosis, thus restoring cellular homeostasis under normal physiological conditions. UV radiation-induced ROS production causes oxidative damage to mitochondrial proteins, lipids, and DNA (mtDNA), leading to mitochondrial dysfunction. Owing to their increased susceptibility to oxidative damage, mitochondria serve as a primary source of ROS. Because mtDNA is attached to the matrix of the mitochondrial inner membrane and is directly exposed to ROS generated by mitochondria, it is highly susceptible to ROS-induced damage. mtDNA does not contain non-coding regions, and mutations caused by oxidative damage may occur during transcription. Under ROS-induced oxidative damage, the likelihood of mutations is 50 times higher in mtDNA than in nuclear DNA.^[59,60]^Furthermore, ROS can directly influence unsaturated lipids present in cardiolipins, leading to their oxidation and conversion into lipid second messengers.^[61]^ Widel et al^[62]^ demonstrated that UVA and UVB radiation induced apoptosis and senescence as a bystander effect in human dermal fibroblasts by stimulating the secretion of ROS and pro-inflammatory cytokines. Dysfunctional mitochondria can produce an increased amount of ROS owing to their high susceptibility to oxidative damage. Excessive ROS production leads to the impairment and irreparability of mitochondria, which subsequently affects mitochondrial membrane potential and permeability through reciprocal mechanisms. The resulting inflammatory responses eventually trigger apoptosis. Altogether, UV radiation disturbs the homeostasis of the skin in vivo, induces oxidative and mitochondrial stress, and exacerbates skin aging, both directly and indirectly (Fig. 1).^[63–65]^

### 3.2. Mechanisms underlying autophagy

**Figure 2.**
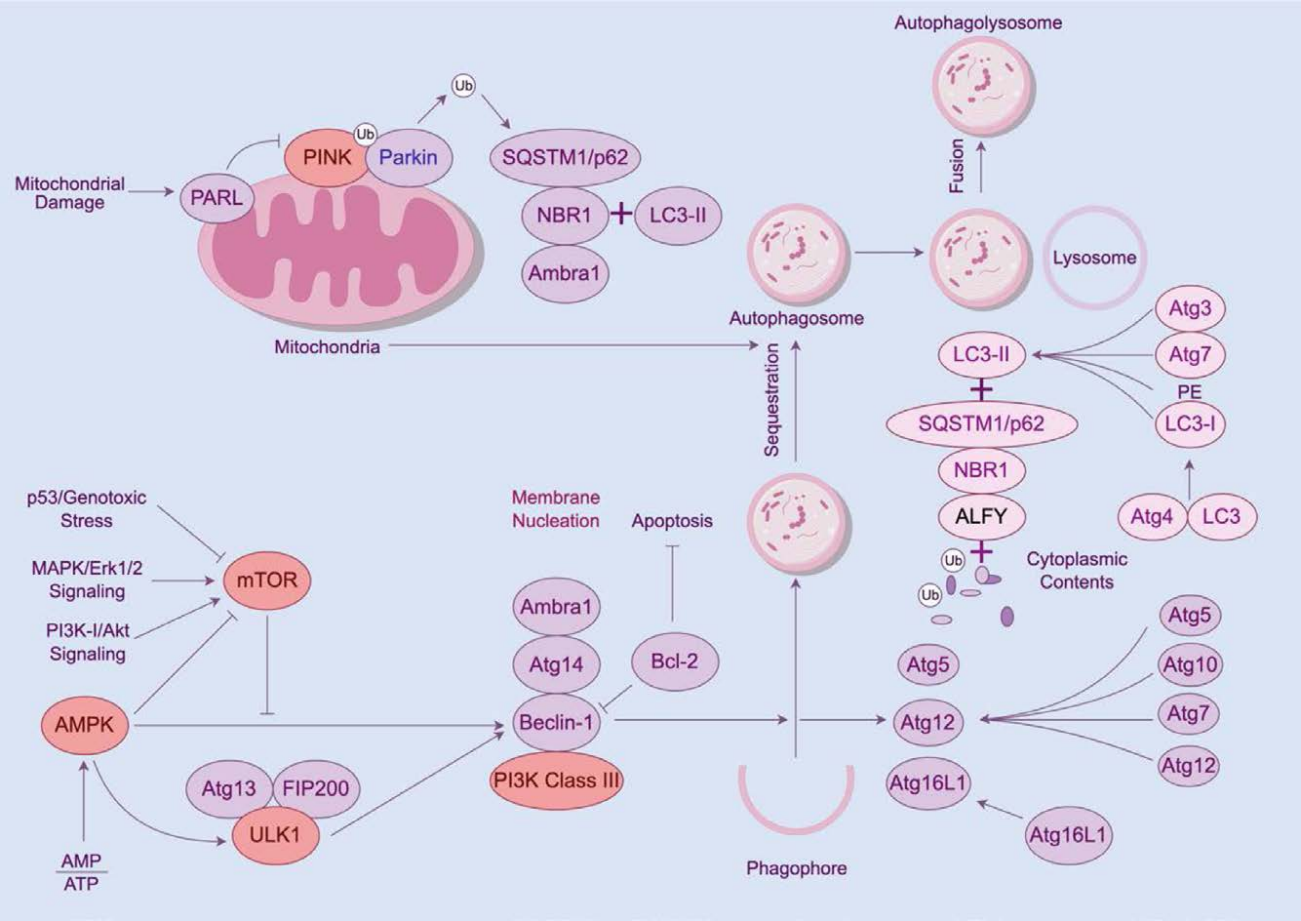
Process of autophagy (drawn using Figdraw). Arrows indicate activating effects and flat heads indicate inhibiting effects. Mitochondrial autophagy occurs through ubiquitin-dependent or ubiquitin-independent pathways.ULK complex (ULK1, ATG13, and FIP200) which is inhibited by mTOR is formed in the presence of cargo encompassing damaged mitochondria. The class III PI3K complex (Atg14, Vps34, and Beclin1) plays a crucial role in autophagosome formation by generating PI3K, which in turn recruits downstream ubiquitin-like conjugation systems (Atg5, Atg12, and Atg16L) and facilitates the conversion of LC3-I to LC3-PE. Then the fusion of lysosomes with autophagosomes forms autolysosomes. Finally, the degraded cargos are digested and the productions are recycled back to cytoplasm by lysosomal hydrolase. Ambra1 = autophagy and Beclin 1 regulator 1, Atg = autophagy-related protein, AMPK = adenosine 5´-monophosphate (AMP)-activated protein kinase, ALFY = autophagy-linked FYVE protein, BNIP3 = Bcl2 interacting protein 3, FIP 200 = FAK family kinase-interacting protein of 200 kDa, LC3 = microtubule-associated light-chain protein 3 1A/1B, mTOR = mammalian target of rapamycin, NBR1 = neighbor of BRCA1 gene 1, PINK1 = PTEN-induced kinase 1,Parkin = E3-ubiquitin (Ub) ligase, PI3K = phosphatidyl inositol 3 kinase, PE = phosphatidylethanolamines, PARL = presenilin associated rhomboid like, SQSTM1/ p62 = adaptor protein sequestosome 1,ULK1 = Unc-51-like autophagy-inhibiting kinase 1.

Approximately 6 decades ago, Deter and De Duve coined the term “autophagy” for the degradation of mitochondria and other intracellular structures within the lysosomes of rat liver.^[66]^ Studies on autophagy initially focused on its role as a stress response mechanism in both yeast and mammalian cells. Autophagy is a cellular process induced under various stress conditions and plays an important role in cellular homeostasis by regulating the normal folding of macromolecules to promote cell survival during starvation, genotoxic stress, and oxidative stress.^[67–69]^ The 3 main types of autophagy are macro-autophagy, molecular chaperone-mediated autophagy, and micro-autophagy.^[70]^ Micro-autophagy involves direct phagocytosis of cargo by lysosomes. Molecular chaperone-mediated autophagy involves the selective recognition of proteins possessing common sequences^[71]^ and their subsequent transport to lysosomal membrane-associated protein 2A through heat shock homologous protein 70.^[72]^ This process facilitates the delivery of unfolded proteins to lysosomes. Macro-autophagy involves the bulk segregation of cytoplasmic cargo into double membrane-bound autophagosomes, which deliver their contents to lysosomes.^[73]^ Autophagy, also known as macro-autophagy, serves as a prominent pathway for the repair of impaired proteins and organelles and the regulation of stress response.^[74]^ Macro-autophagy has been shown to play a predominant role in preventing skin photoaging. therefore, autophagy discussed in this study refers specifically to macro-autophagy. Autophagy is involved in various physiological and pathological processes within an organism, including development,^[75]^ tumor suppression,^[76–78]^ antigen presentation,^[79,80]^ cellular survival,^[81]^ programmed cell death,^[82,83]^ inflammatory balance,^[84]^ and aging.^[85,86]^ It primarily functions as a cellular protective mechanism that requires rigorous regulation. Under normal physiological conditions, cells have low basal autophagic activity; however, under pathological conditions, external or internal stress stimuli may lead to a rapid increase in autophagic activity. Consequently, redox dysregulation reduces autophagic activity. To date, several studies have attempted to investigate the mechanisms underlying the regulation of autophagy. Based on the identified mechanisms, autophagy is classified as ubiquitin-dependent and ubiquitin-independent. Ubiquitin-dependent autophagy is primarily regulated by PTEN-induced kinase 1 (PINK) and Parkin RBR E3 ubiquitin-protein ligase (PARKIN), whereas ubiquitin-independent autophagy is regulated by mitochondrial autophagic receptors that can directly bind to microtubule-associated protein 1A/1B-light chain 3 (LC3) without inducing extensive ubiquitination (Fig. 2).^[69,74,87]^ Macrophage autophagy is divided into 3 phases, namely, phagocytosis initiation, extension, and cargo degradation.^[58]^ The initiation phase of autophagy involves the interaction between the Unc-51-like kinase (ULK1) complex formed by 3 proteins (ULK1, FAK family kinase-interacting protein of 200 kDa, and autophagy-related protein 13) and the mammalian target of rapamycin complex 1 (mTORC1). mTORC1 plays a key role in regulating autophagy,^[88]^ cell growth and proliferation, and protein synthesis. It is regulated by the class III phosphatidylinositol-3-kinase pathway and its negatively regulated phosphatases and tropomyosin homologs. When mTORC1 activity is inhibited under nutrient-deficient conditions, adenylate-activated kinase phosphorylates and binds to ULK1^[89]^ and interacts with 200-kDa family-interacting proteins to induce the nucleation and elongation of autophagosomes.^[90]^ Two ubiquitin-like conjugation systems (conjugation of ATG8 protein to phosphatidylethanolamine and conjugation of ATG12 to ATG5) play important roles in the elongation step.^[91]^ Atg12 binds to autophagy-related protein 16 (Atg16) through the activation of E1-like enzyme and transport of E2-like enzyme.^[92]^ LC3 produces soluble LC3-I through the action of Atg4, and LC3-I subsequently forms a complex with phosphatidylethanolamine LC3-II through the action of E1-like enzyme and E2-like enzyme. Because LC3-II participates in autophagosome elongation until autophagic lysosomes are formed, it is considered a marker of autophagy.^[11,93]^ The final stage of autophagy involves the formation of autophagic lysosomes through the fusion of autophagosomes and lysosomes.^[94]^ A reduction in mitochondrial membrane potential can stabilize PINK1, which is ubiquitinated after the recruitment of mitochondrial PARKIN,^[87]^voltage-dependent anion channels,^[95]^ or other mitochondrial proteins. Modified mitochondria are recognized by sequestosome 1^[96]^ or other factors and transported to autophagosomes for degradation.

### 3.3. Autophagy and photoaging

#### 3.3.1. Autophagy regulates UV-induced oxidative stress.

In daily life, humans are constantly exposed to oxidants resulting from either endogenous metabolic processes or exogenous environmental pollution. Prolonged exposure to UV radiation, particularly UVA and UVB, triggers a significant increase in the production of ROS in skin cells. Consequently, the imbalance between oxidant and antioxidant mechanisms leads to mitochondrial impairment and oxidative stress. As a result, the human body produces a large number of oxidative byproducts, including free radicals. Activated PINK1 recruits PARKIN to the surface of damaged mitochondria, resulting in autophagy.^[11,92]^ Low levels of oxidative stress can enhance mitochondrial biogenesis and promote the elimination or repair of damaged mitochondria. On the contrary, high levels of oxidative stress suppress the ability of the cell to alleviate oxidative damage, consequently resulting in the accumulation of damaged mitochondria.^[97,98]^ Under skin photoaging, autophagy is activated in response to the excessive production of ROS. Upon activation, autophagy breaks down and eliminates oxidized lipids and proteins and prevents the accumulation of ROS. Consequently, metabolic homeostasis is restored, and oxidative damage is alleviated, decelerating the onset of photoaging. However, excessive accumulation of ROS inhibits autophagy and decreases its clearance efficiency in photoaging skin cells.^[99]^ These effects, in turn, exacerbate oxidative damage and contribute to the development of roughness, wrinkles, hyperpigmentation, and other manifestations of photoaging.^[14,100]^ Prolonged exposure to UVB radiation leads to the synthesis of interleukin-1α, which triggers the release of granulocyte–macrophage colony-stimulating factor.^[101]^ These 2 factors subsequently penetrate the dermis and activate fibroblasts, resulting in the production of neutral lysozyme, which cleaves and destroys the elastic fiber network encapsulating fibroblasts, thus decreasing skin elasticity and causing wrinkles.^[101]^ UVA radiation penetrates the dermis and stimulates the expression of MMP-1 (type I collagenase) and the secretion of interleukin-6, leading to sagging of the skin.^[102]^ Lim et al^[103]^ found that α-neoendorphin activated autophagy and inhibited ROS production induced by UVB radiation. These changes decreased the production of skin photoaging-related factors and the activity of MMPs and increased the synthesis of procollagen, eventually alleviating the damage caused by UVB-induced photoaging. Zheng et al^[104]^ showed that isoorientin exerted protective effects on human dermal fibroblasts irradiated with UVB by enhancing cell viability, suppressing the expression of MMP1 and MMP3 (type III collagenase), inhibiting oxidative stress, and inducing autophagy.

#### 3.3.2. Autophagy regulates UV-induced DNA damage.

Various pharmacological and environmental factors, including UV radiation, can induce genotoxicity, leading to direct or indirect DNA damage.^[105]^ UVA radiation indirectly causes oxidative damage to DNA by inducing the formation of cyclobutane pyrimidine dimers (CPDs) through excessive production of ROS in the skin, consequently leading to DNA fragmentation.^[106]^ UVB radiation is directly absorbed by the DNA of epidermal cells, resulting in the formation of CPDs and other photoproducts.^[107]^ UVA-induced indirect DNA damage is mainly repaired through base excision repair, whereas UVB-induced direct damage is repaired through nuclear excision repair.^[108]^Studies have shown that exposure of the skin to UVB radiation activates adenosine 5´-monophosphate (AMP)-activated protein kinase,^[109,110]^ thereby inducing autophagy. Subsequently, activation of autophagy increases the levels of the damage sensor protein XPC and promotes the repair of CPDs, positively regulating the repair of DNA damage.^[111]^ In addition, UVA-induced indirect DNA damage can stimulate the autophagy receptor protein p62, which promotes the repair of DNA damage through base excision repair.^[112]^ Umar et al^[108]^ demonstrated that increased levels of autophagy alleviated nuclear DNA damage and promoted the repair process in human primary dermal fibroblasts exposed to UVB radiation. Consistently, Song et al^[113]^ demonstrated that exposure to both internal and external stimuli exacerbated DNA damage in cells lacking Atg7. These studies suggest that regulating autophagy to release DNA damage is a promising strategy for delaying the onset of skin photoaging.

#### 3.3.3. Role of autophagy in UV-induced cell proliferation and apoptosis.

Autophagy which is tightly controlled primarily avoids excessive degradation of the cellular content, whereas apoptosis eventually results in cell death. These 2 pathways are regulated by shared factors and modulate each other’s activities. Exposure of the skin to UV radiation leads to several deleterious effects, including disruption of cellular metabolism, morphological and structural changes, and alterations in the differentiation, proliferation, and apoptosis of skin cells.^[114]^ Under normal physiological conditions, compensatory over-proliferation is initiated to replenish skin cells after apoptosis and maintain in vivo homeostasis.^[115]^ Basal levels of autophagy ensure the physiological rejuvenation of senescent and dysfunctional organelles. Autophagy regulates the cell fate through various intercellular communication signals. The mechanisms underlying UV-induced cell proliferation and apoptosis are tightly controlled to prevent the oncogenic expansion of UV-damaged cells. UVA and UVB radiation can induce apoptosis in epidermal cells by increasing the expression of the tumor suppressor protein p53 and Bcl-2 associated X, decreasing the expression of B-cell lymphoma-2,^[116]^ and activating p38 mitogen-activated protein kinase. Ultraviolet radiation-associated gene regulates autophagy by promoting the formation and maturation of autophagosomes. In addition, it inhibits the transport of Bcl-2 associated X from the cytoplasm to the mitochondria during apoptosis induced by UV radiation in human tumor cells.^[117]^ Cyclooxygenase 2 is another important mediator of UV-induced cell proliferation and apoptosis. Both UVA and UVB radiations can upregulate Cyclooxygenase 2,^[118]^ which catalyzes the rate-limiting step in the synthesis of prostaglandin E2. Prostaglandin E2 signals through autocrine and paracrine mechanisms to promote cell proliferation and inhibit apoptosis. In addition, the mammalian target of rapamycin (mTOR) signaling pathway regulates cell growth and proliferation in response to stress.^[119]^ Sestrin 2, a stress-inducible protein that is activated upon UVB exposure,^[118]^inhibits cell proliferation through negative regulation of mTOR signaling.^[116]^ Similarly, adenosine 5´-monophosphate (AMP)-activated protein kinase is an inhibitor of mTOR signaling and inhibits cell proliferation in response to UVB exposure.^[120]^ UV radiation induces cell proliferation and apoptosis to maintain tissue homeostasis. UVB radiation induces autophagy to decrease p62 levels to prevent p62-mediated activation of p38 mitogen-activated protein kinase and inhibit subsequent apoptosis.^[112]^ In addition, it stimulates the autophagy activator Sestrin 2 to promote cell survival. Parrado et al^[121]^ demonstrated that Polypodium leucotomos extract effectively served as a photoprotective agent, mitigating the detrimental effects of apoptosis and necrosis induced by UV radiation. Additionally, it inhibited abnormal extracellular mesenchymal remodeling, eventually suppressing cell proliferation induced by UV radiation. Wondrak et al^[122]^ reported that photosensitization by both endogenous and synthetic 3-hydroxypyridine derivatives inhibited the proliferation of cultured human skin keratinocytes and fibroblasts and induced their apoptosis in a dose-dependent manner. In an independent study, Li et al^[123]^ found that cannabinol effectively inhibited UVB-induced cytotoxicity, apoptosis, and G2/M cell cycle arrest in human keratinocytes by modulating autophagy. In addition, cannabinol counteracted aberrant cell proliferation in UVB-exposed photodamaged mouse skin and suppressed the expression of cyclooxygenase-2 protein, thereby improving the overall skin condition.

## 4. Conclusion

**Figure 3.**
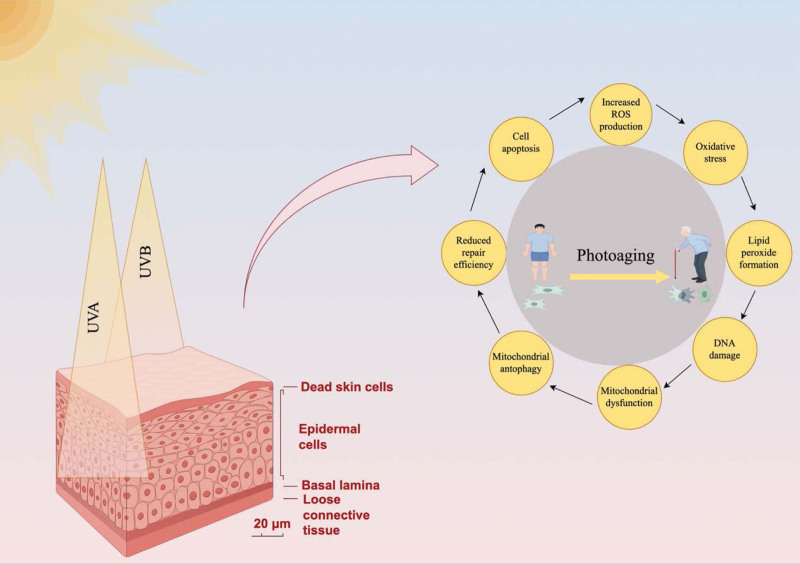
Role of autophagy in skin photoaging (drawn using Figdraw). Arrows indicate activating effects. The unique physical attributes of solar radiation facilitate its penetration into the skin and its interaction with cells at different depths, resulting in distinct and interconnected biological responses. ROS = reactive oxygen species, UVA = ultraviolet A, UVB = ultraviolet B.

Skin photoaging occurs owing to exogenous factors and is considered the most easily identifiable type of aging. In recent years, the incidence of photoaging and UV-related skin cancer has gradually increased worldwide. As individuals pursue a better lifestyle in this new era, they are increasingly focusing on maintaining both aesthetic and hygiene. Therefore, developing effective strategies for the prevention and treatment of photoaging is necessary. During UV-induced photodamage, autophagy activates relevant signaling pathways to regulate apoptosis, facilitate the repair of DNA damage, and promote the removal of oxidized lipids, consequently delaying the onset of skin photoaging (Fig. 3).^[87]^ Therefore, regulating autophagy is considered a novel strategy for preventing skin photoaging. However, the mechanisms through which autophagy delays skin photoaging remain elusive. Further studies should aim to develop autophagy-targeted strategies for the prevention and treatment of UV-induced skin photoaging.

## Acknowledgments

We thank Bullet Edits Limited for the linguistic editing and proofreading of the manuscript.

## Author contributions

**Conceptualization:** Xiaojiao Zhong, Ying Deng, Hongqiu Yang, Xiaoshuang Du, Ping Liu.

**Funding acquisition:** Yu Du.

**Methodology:** Xiaojiao Zhong, Ying Deng, Hongqiu Yang.

**Software:** Xiaojiao Zhong, Ying Deng.

**Supervision:** Yu Du.

**Writing – original draft:** Xiaojiao Zhong.

**Writing – review & editing:** Yu Du, Ying Deng.
